# Differential Recognition of Terminal Extracellular *Plasmodium falciparum* VAR2CSA Domains by Sera from Multigravid, Malaria-Exposed Malian Women

**DOI:** 10.4269/ajtmh.14-0524

**Published:** 2015-06-03

**Authors:** Mark A. Travassos, Drissa Coulibaly, Jason A. Bailey, Amadou Niangaly, Matthew Adams, Myaing M. Nyunt, Amed Ouattara, Kirsten E. Lyke, Matthew B. Laurens, Jozelyn Pablo, Algis Jasinskas, Rie Nakajima, Andrea A. Berry, Shannon Takala-Harrison, Abdoulaye K. Kone, Bourema Kouriba, J. Alexandra Rowe, Ogobara K. Doumbo, Mahamadou A. Thera, Miriam K. Laufer, Philip L. Felgner, Christopher V. Plowe

**Affiliations:** Howard Hughes Medical Institute/Center for Vaccine Development, University of Maryland School of Medicine, Baltimore, Maryland; Malaria Research and Training Center, University of Sciences, Techniques and Technology, Bamako, Mali; Division of Infectious Diseases, Department of Medicine, University of California, Irvine, California; Centre for Immunity, Infection and Evolution, Institute of Immunology and Infection Research, School of Biological Sciences, University of Edinburgh, Edinburgh, United Kingdom

## Abstract

The *Plasmodium falciparum* erythrocyte membrane protein 1 (PfEMP1) family mediates parasite sequestration in small capillaries through tissue-specific cytoadherence. The best characterized of these proteins is VAR2CSA, which is expressed on the surface of infected erythrocytes that bind to chondroitin sulfate in the placental matrix. Antibodies to VAR2CSA prevent placental cytoadherence and protect against placental malaria. The size and complexity of the VAR2CSA protein pose challenges for vaccine development, but smaller constitutive domains may be suitable for subunit vaccine development. A protein microarray was printed to include five overlapping fragments of the 3D7 VAR2CSA extracellular region. Malian women with a history of at least one pregnancy had antibody recognition of four of these fragments and had stronger reactivity against the two distal fragments than did nulliparous women, children, and men from Mali, suggesting that the C-terminal extracellular VAR2CSA domains are a potential focus of protective immunity. With carefully chosen sera from longitudinal studies of pregnant women, this approach has the potential to identify seroreactive VAR2CSA domains associated with protective immunity against pregnancy-associated malaria.

## Introduction

*Plasmodium falciparum*, the deadliest and most common species of malaria in Africa, has the ability to adhere to capillary beds due to proteins expressed on the surface of infected red blood cells. During pregnancy, this sequestration of infected erythrocytes and the ensuing inflammatory response reduce placental blood flow and lead to fetal and maternal compromise. Naturally acquired immunity to malaria develops in childhood and appears, in part, to be related to the development of antibodies to these malaria parasite surface antigens. The *var* family of genes encodes one such set of antigens, *P. falciparum* erythrocyte membrane protein 1 (PfEMP1), which bind to endothelial receptors.^1^ Each parasite genome carries about 60 *var* genes but expresses only one predominant PfEMP1 at a time, providing a large repertoire of surface molecules believed to be important for immune evasion and pathogenesis. In pregnant women, parasitized erythrocytes expressing the PfEMP1 VAR2CSA bind to chondroitin sulfate A (CSA) in the placental matrix,^2^–^4^ leading to chronic infection and inflammation. Thus, although adults achieve a state of semi-immunity in which they are protected from severe disease and death due to *P. falciparum*, pregnant women have an increased vulnerability to infection, especially during their first or second pregnancy, because they lack a protective immune response to this antigen. With each pregnancy, women develop antibodies that protect against placental adhesion of infected erythrocytes and are associated with improved birth outcomes.^5^

The size and complexity of the VAR2CSA protein pose challenges for vaccine development, but smaller constitutive domains may be suitable for a subunit vaccine targeted against pregnancy-associated malaria. Studies attempting to identify which of the seven constitutive extracellular VAR2CSA domains are critical to the protective antibody response have produced differing results,^6^,^7^ in part due to different protein expression systems and serum sample comparisons. Current pregnancy-associated malaria vaccine development efforts have focused on the first four extracellular VAR2CSA domains, given their role in CSA binding.^8^–^10^ When probed with appropriately expressed VAR2CSA constitutive domains, sera from women with at least one pregnancy should react to more VAR2CSA domains as well as domains critical to the protective antibody response more strongly than sera from nulliparous women, men, and children. To determine whether such differential seroreactivity of VAR2CSA fragments exists, reactivity to constitutive domains of VAR2CSA was measured with a protein microarray using sera from children, men, and women living in an area with highly seasonal but intense malaria transmission in Mali, west Africa.

## Materials and Methods

Serum samples were obtained from 75 children aged 1–6 years and 77 adults, including 32 men and 45 women enrolled in malaria vaccine trials conducted in Bandiagara, Mali.^11^–^13^ Sixteen women reported that they were never pregnant, while the remaining 29 women had between one to nine previous pregnancies, with a median of three pregnancies. The median age of women was 27 years, while the median age of men was 23 years. Sera from 11 malaria-naive U.S. blood donors were used as controls. Vaccine trials were conducted in compliance with the International Conference on Harmonisation Good Clinical Practices, the Declaration of Helsinki, and Malian regulatory requirements.

**Figure 1.**
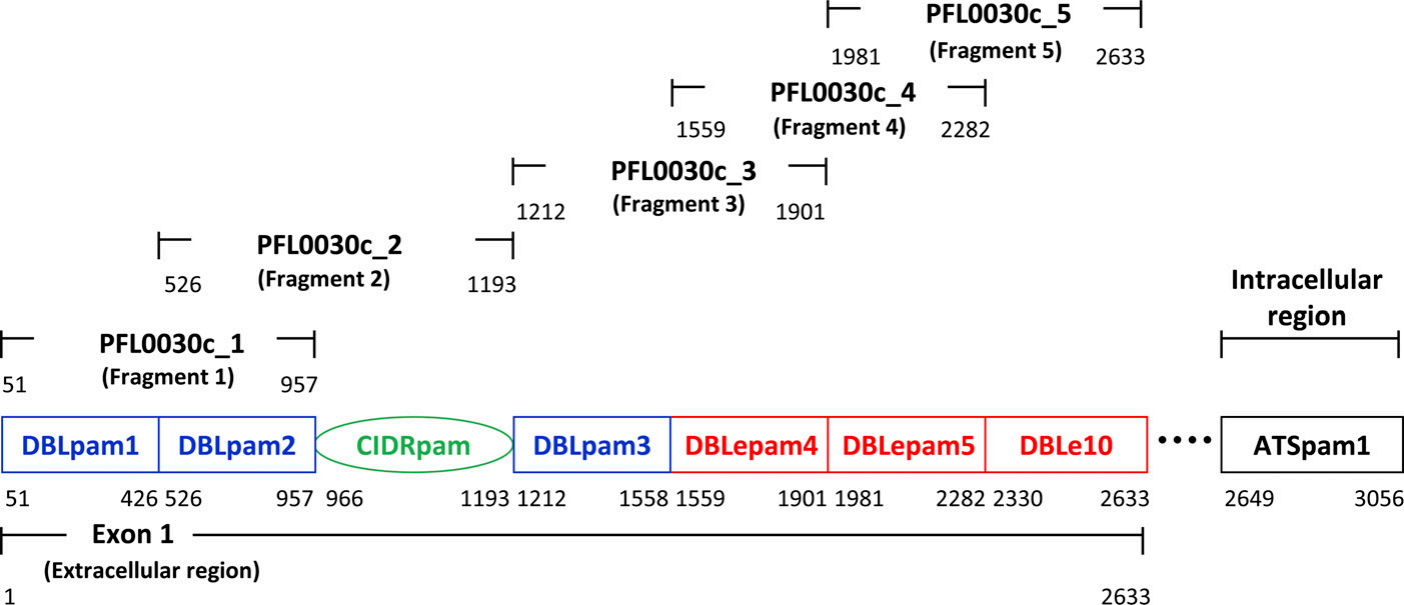
VAR2CSA fragments on the microarray included all seven extracellular domains of PFL0030c, the VAR2CSA in the 3D7 reference genome of *Plasmodium falciparum*. Starting and ending amino acid positions are indicated below each fragment and domain.

A protein microarray was designed to include five 3D7-based VAR2CSA fragments (Figure 1). For simplicity, PFL0030c_1, _2, _3, _4, and _5 are henceforth referred to as Fragments 1, 2, 3, 4, and 5, respectively. Start and endpoints of constitutive VAR2CSA domains were based on a recent reclassification of *var* domains.^14^ Each fragment was designed to include two consecutive extracellular constitutive domains. Array construction occurred as previously described, including transcription and translation using a cell-free *Escherichia coli*-based in vitro system,^15^–^17^ with the addition of the RTS 100 *E. coli* Disulfide kit (5 Prime, Gaithersburg, MD) to support disulfide bond formation. Protein microarray printing and quality control protocols have been previously described.^18^ Arrays were probed with 2 μL of participant sera after a 1-hour incubation with *E. coli* lysate to bind nonmalaria-specific antibodies in each sample. Probing and scanning protocols have been detailed elsewhere.^18^ After overnight antibody hybridization at 4°C, arrays were washed, stained, and dried before scanning with a microarray scanner (PerkinElmer, Waltham, MA).

Raw signal intensity was reduced by the mean of the “no-DNA”-negative controls to determine fluorescence intensity. Samples with negative fluorescence intensity were treated as zero fluorescence intensity. “Recognition” of a VAR2CSA fragment was defined as fluorescence intensity significantly greater than that of the malaria-naïve control group, based on a two-sample Kolmogorov–Smirnov test. All other unmatched fluorescence intensity comparisons between groups were likewise made with a two-sample Kolmogorov–Smirnov test. These statistical analyses were performed using MYSTAT 12, Version 12.02.00 (Systat Software, Inc., San Jose, CA). Multivariate analysis was conducted using linear regression modeling with SAS 9.3.

## Results

**Figure 2.**
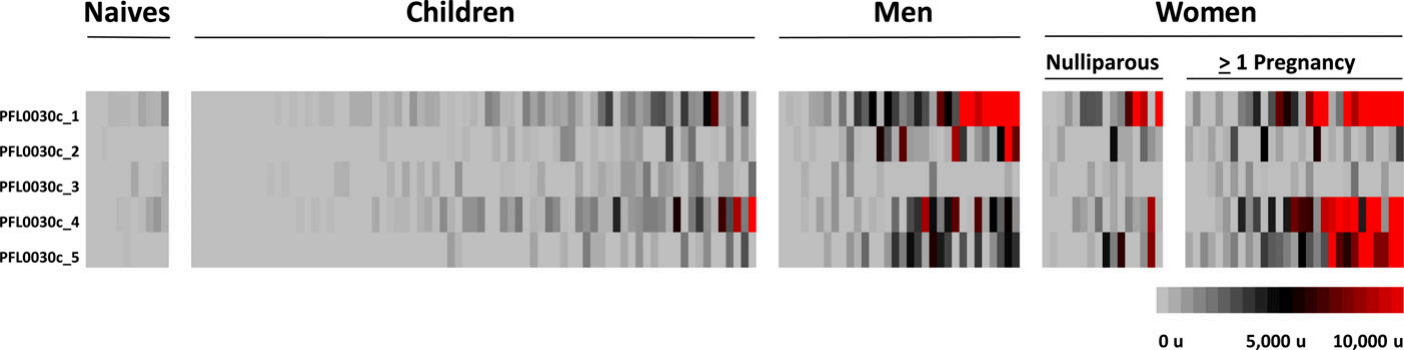
Heat map of seroreactivity to five 3D7 VAR2CSA fragments, with each fragment separated by row. Each column displays the profile of one serum sample. Grey color indicates no seroreactivity, black is low-to-moderate seroreactivity, and red denotes high seroreactivity to probed fragments.

Women with at least one pregnancy had antibody recognition to four out of five VAR2CSA fragments (Fragment 1: *P* = 0.002; Fragment 2: *P* = 0.004; Fragment 4: *P* < 0.001; Fragment 5: *P* < 0.001; Figure 2). Men had antibody recognition to three VAR2CSA fragments (Fragment 1: *P* = 0.001; Fragment 2: *P* < 0.001; Fragment 5: *P* = 0.003). Although some men also reacted to Fragment 4, as a group, men did not significantly recognize this fragment more than controls (*P* = 0.065). In contrast, nulliparous women and children did not have antibody recognition to any VAR2CSA fragments. A few nulliparous women recognized Fragment 1 in particular, but overall, they did not significantly recognize this fragment more than controls (Fragment 1: *P* = 0.066). Fragment 3 was not recognized by any group (*P* > 0.15).

**Figure 3.**
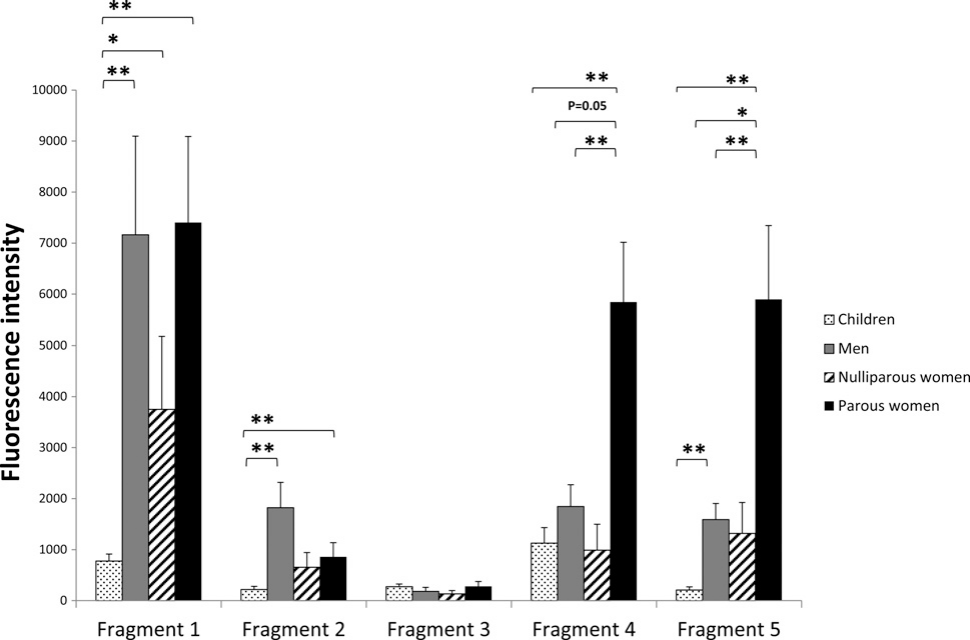
Average fluorescence intensities of subjects for each VAR2CSA fragment. Values are reported as means with standard errors. *P < 0.05; **P < 0.01. Two-sample Kolmogorov–Smirnov test.

Women with at least one pregnancy recognized two fragments, Fragments 4 and 5, more strongly than any other malaria-exposed groups (Figure 3), including nulliparous women (Fragment 4: *P* = 0.004; Fragment 5: *P* = 0.002), men (Fragment 4: *P* = 0.052; Fragment 5: *P* = 0.040), and children (Fragment 4: *P* < 0.001; Fragment 5: *P* < 0.001). A multivariable linear regression model for predicting fluorescence intensity found a significant association with numbers of pregnancies, controlling for age, for both Fragment 4 (*P* = 0.02) and Fragment 5 (*P* = 0.04), but not for other VAR2CSA fragments (Fragment 1: *P* = 0.51; Fragment 2: *P* = 0.27; Fragment 3: *P* = 0.85). There was no association of fluorescence intensity with age when controlling for numbers of pregnancies (Fragment 1: *P* = 0.94; Fragment 2: *P* = 0.53; Fragment 3: *P* = 0.85; Fragment 4: *P* = 0.14; Fragment 5: *P* = 0.51).

All three groups of malaria-exposed adults had higher antibody recognition of Fragment 1 than did children (nulliparous women: *P* = 0.045; women with at least one pregnancy: *P* < 0.001; men: *P* < 0.001). Sera from the three groups of adults did not differ in recognition of this fragment (women with at least one pregnancy versus nulliparous women: *P* = 0.265; women with at least one pregnancy versus men: *P* = 0.737; nulliparous women versus men: *P* = 0.341).

In contrast to nulliparous women, women with at least one pregnancy and men had higher antibody recognition of Fragment 2 than did children (nulliparous women: *P* = 0.181; women with at least one pregnancy: *P* = 0.004; men: *P* < 0.001).

## Discussion

A microarray with five 3D7 VAR2CSA fragments was probed with sera from adults and children living in a malaria-endemic region, revealing differential seroreactivity by age, sex, and number of pregnancies. Sera from women with at least one pregnancy recognized more VAR2CSA fragments than sera from nulliparous women, men, and children. Sera from multiparous women recognized the two C-terminal fragments, Fragments 4 and 5, to a greater extent than sera from any other group. Moreover, increased gravidity was associated with higher seroreactivity to these two fragments.

Fragments 4 and 5 together spanned the last three constitutive domains of the extracellular portion of the 3D7 VAR2CSA, and both fragments included the DBLepam5 domain. Higher levels of antibodies against DBLepam5 have been found in plasma from pregnant Senegalese women than in plasma from men or nulligravid women.^19^ In Malawi, sera from multigravid women recognized this domain more strongly than sera from primigravid women.^20^ However, other studies have not supported the finding of DBLepam5 as the VAR2CSA domain primarily recognized by antibodies from multigravid women. Antibodies to the DBLepam5 domain have been detected in sera from young Tanzanian children^21^ and Colombian men and children.^22^ Antibody recognition of other individual VAR2CSA domains has correlated with gravidity, including DBLpam1, DBLpam3, and DBLe10.^7^,^19^ Antibody recognition to no single VAR2CSA domain has been associated with absence of placental malaria.^23^ Our approach differs from these studies in the use of a cell-free *E. coli* system, the coupling of consecutive VAR2CSA domains to account for domain–domain interactions that may contribute to immune recognition, and sampling from a diverse set of malaria-exposed subjects, including different ages, sexes, and gravidities. The high throughput capacity of a protein microarray further strengthens this multidomain approach to understanding the development of VAR2CSA immunity.

Seroreactivity studies of VAR2CSA have typically compared serum results from pregnant women. Women included in this study were not pregnant at the time of serum collection, but antibody responses to VAR2CSA were nonetheless robust. However, this difference in serum sampling may account for some discrepancies with results from previous studies if, for example, transient rises in antibody levels to particular VAR2CSA domains occur during pregnancy. A limitation of this study is that some VAR2CSA domains may not have been folded correctly with the protein expression system used. This may have been the case with Fragment 3, a fragment for which there was no significant seroreactivity, suggesting that it may not have been expressed appropriately, particularly given that previous work has found that the two VAR2CSA domains included in this fragment, DBLpam3 and DBLpam4, were seroreactive in multigravid east African women.^7^

Fragments 1 and 2 were recognized significantly more in adults than in children, but not significantly more in women with at least one pregnancy. This suggests that greater malaria exposure leads to increased recognition of these two fragments, possibly due to their similarity to other malaria antigens or to a secondary, as yet undetermined role of VAR2CSA in which specific VAR2CSA regions within these fragments are exposed to the immune system. Both Fragments 1 and 2 contained most of the minimal CSA-binding region ID1-DBL2pam-ID2a. A recent Cameroon study also found that seroreactivity to the CSA-binding region was not associated with gravidity.^24^ Indeed, Cameroonian men reacted just as much to this region as did Cameroonian women, suggesting an association with malaria exposure similar to our findings.

Other studies investigating PfEMP1 seroreactivity have used small segments, typically less than 300 amino acids, of single domains such as the first DBL-alpha domain.^25^–^27^ These small fragments may fail to react with antibodies that bind to discontinuous epitopes contained outside their boundaries. Here, we cloned domains as overlapping pairs and observed a high level of reactivity against these larger fragments. This approach is inclusive of interdomain and discontinuous epitopes that may improve sensitivity. A subtractive approach can then be used to map the actual antibody binding sites.

Although expression of the entire VAR2CSA extracellular region may be impractical for vaccine development, our findings suggest that focusing efforts on the last three constitutive extracellular domains may produce seroreactivity similar to that observed in multigravid women from a malaria-endemic region. This contrasts with current pregnancy-associated malaria vaccine development efforts that have focused on the first four extracellular VAR2CSA domains, given their role in CSA binding.^8^–^10^ In addition, expression of consecutive VAR2CSA domains may produce seroreactivity results that more accurately reflect acquisition of natural immunity than single VAR2CSA domains alone.

Protein microarrays are a powerful tool to identify PfEMP1 domains uniquely seroreactive to multigravid women in malaria-endemic regions, thus shedding further light on the acquisition of natural immunity to pregnancy-associated malaria. Microarrays have been successfully populated with multiple PfEMP1 variants^17^ and field variants of other malaria vaccine candidate antigens.^28^ Our next step is to develop a microarray with fragments from multiple, diverse VAR2CSA variants derived from laboratory strains and field isolates that can be probed with serum samples from malaria-endemic regions, particularly from longitudinal studies that include pregnant women, to understand the role of VAR2CSA diversity in protective immunity. We will compare seroreactivity of individual VAR2CSA domains versus coupled consecutive domains to determine which fragments best reflect the development of protective immunity and compare results to current pregnancy-associated malaria vaccine candidates, including variants of the minimal CSA-binding region ID1-DBL2pam-ID2a. VAR2CSA field variants will be an important component of this second generation array, given the extraordinary genetic diversity of *var* genes,^14^ which may not be adequately represented in laboratory strains. This approach may provide additional insight into the development of pregnancy-associated antimalarial immunity and the need for multiple VAR2CSA domain targets for a vaccine that would potentially be efficacious against pregnancy-associated malaria in diverse geographic regions.
